# Survival outcomes of patients with oligometastatic non-small cell lung cancer who were treated with radical therapy: a multicenter analysis

**DOI:** 10.55730/1300-0144.5659

**Published:** 2023-02-01

**Authors:** Özgür AÇIKGÖZ, Ahmet BİLİCİ, Deniz TATAROĞLU ÖZYÜKSELER, Sabin GÖKTAŞ AYDIN, Fatih SELÇUKBİRİCİK, Rashad RZAZADE, Ömer Fatih ÖLMEZ, Hale BAŞAK ÇAĞLAR

**Affiliations:** 1 Department of Medical Oncology, Faculty of Medicine, İstanbul Medipol University, İstanbul, Turkiye; 2 Department of Medical Oncology, Dr. Lütfi Kırdar Kartal Education and Research Hospital, İstanbul, Turkiye; 3 Department of Medical Oncology, Faculty of Medicine, Koç University, İstanbul, Turkiye; 4 Department of Radiation Oncology, Anadolu Health Center, Kocaeli, Turkiye

**Keywords:** Oligometastasis, nonsmall cell lung cancer, radical treatment, targeted therapy

## Abstract

**Background/aim:**

Oligometastatic disease for nonsmall cell lung cancer (NSCLC) patients is generally thought to represent a better prognosis with a quieter biology, limited number of disease sites and long-term disease control. In this study, we aimed to determine the efficacy of radical treatment options for patients with oligometastatic NSCLC.

**Materials and methods:**

This retrospective trial included totally 134 patients with oligometastatic NSCLC. The presence of oncodriver mutation, tumor stages and nodal status, the number of metastases and involved metastatic site, treatment of primary tumor and oligometastasis, response rate, overall survival (OS) and progression-free survival (PFS) were evaluated.

**Results:**

Of 134 patients 66.4% were defined as adenocarcinoma, 26.1% were squamous cell carcinoma and 7.5% of patients were in other histology. Based on the treatment of primary tumor, in 36 patients (26.9%) curative surgery has undergone, in addition, 19 (14.2%) patients were received chemotherapy, 73 (54.5%) were treated with chemoradiotherapy, while immunotherapy and targeted therapy were used in 1 (0.7%) and 2 (1.4%), respectively. The preferred treatment for oligometastatic lesions were SBRT in 72.4% of patients, surgery in 10.5%, and both SBRT and surgery in 17.1% of patients. At the median follow up of 31.3 months (range: 9.5–48.5), the median PFS and OS times were 17 and 24.4 months, respectively. Moreover, OS-2 after progression was also 7.2 months.

**Conclusion:**

Based on our real-life experience, we demonstrated a significant correlation between good response to first treatment and survival in oligometastatic disease, we also understand that local ablative treatment modalities prolong and also delay both OS and PFS in oligometastatic NSCLC patients OS-2.

## 1. Introduction

Lung cancer is the most commonly diagnosed cancer for both sexes, and the leading cause of cancer death among males [1]. Nonsmall cell lung cancer (NSCLC) accounts for the majority of all lung cancer cases. In addition, most cases of NSCLC are diagnosed at an advanced stage [2]. In these patients and those who have relapsed after prior curative treatment, the standard treatment is systemic therapy including immunotherapy, platinum-based doubled chemotherapy, and targeted therapies according to both the driver-mutation and programmed death-ligand 1 (PD-L1) expression status. Some advanced-stage NSCLC patients have limited and organ site metastasis, which is called oligometastasis. The term “oligometastatic disease” was first introduced by Hellman and Weichselbaum [3] 26 years ago, and has become very meaningful in terms of treatment and clinical course ever since. Control of both primary lung lesion and metastatic lesion with local treatments may positively affect overall survival (OS) [4].

Oligometastatic disease for NSCLC patients is generally thought to represent a better prognosis with a less aggressive pathology, a limited number of disease sites and long-term disease control. The better survival outcomes for oligometastatic disease might be associated with the effectiveness of positron emission tomography (PET) staging, well-tolerated cytotoxic agents, molecular targeted therapy and immunotherapy [4, 5].

It was first seen in adding liver metastasectomy for patients with colorectal cancer that aggressive treatment options control oligometastatic diseases in long term [6, 7]. However, other local ablative treatments may be used in limited area oligometastatic diseases. Treatments for patients with cranial and extracranial metastases may include surgery, stereotactic body radiation therapy (SBRT), stereotactic radiosurgery (SRS), radiofrequency ablation (RFA) and microwave ablation [8].

The most important factor that determines the prognosis in oligometastatic NSCLC depends on the synchronous and metachronous detection of metastases [9]. From the large number of retrospective trials, it may be inferred that, at least in specialized centers, radical treatment approaches for oligometastases are widely used. A recent metaanalysis of aggressive treatment of oligometastatic NSCLC based on data from 757 individual patients revealed several prognostic factors. Among other determinants, a significantly worse outcome in OS for the synchronous than the metachronous appearance of oligometastases was reported in that metaanalysis [10].

The adrenal glands, brain and bones are the most common metastatic sites in NSCLC patients. The most common metastasis among these is the brain. Survival results with radical therapy are good in patients with 1–3 metastases. In one study, it was shown that the treatment of solitary brain metastases with surgery or whole-brain radiation therapy (WBRT) offers a 5-year survival advantage (median 36.8% and 20.3 months, respectively) [11]. In addition, local ablative treatments may be preferred in patients with oligometastatic NSCLC with unilateral adrenal gland metastasis [12]. In unilateral adrenal gland metastasis, OS with laparoscopic surgery may vary depending on whether the metastasis is synchronous or metachronous (median 12 vs. 31 months and 5-year OS rates of 25% and 26%, respectively) [13]. Novel RT techniques now provide a more comfortable local ablative treatment than conventional RT techniques. Scorsetti et al. demonstrated the efficacy of SBRT for adrenal metastasis based on retrospective data, which included 64% (18/28 cases) NSCLC patients. They reported that the median OS time was 22.8 months and the 2-year OS rate was 53% [14].

In the current study, we aimed to determine the efficacy of local ablative treatment options for patients with oligometastatic NSCLC.

## 2. Materials and methods

In this study, patients with oligometastatic NSCLC who were treated with radical treatments between 2016 and 2020 were retrospectively analyzed. Demographic characteristics such as age, sex, smoking history, ECOG performance score, histopathologic subtypes, the presence of oncodriver mutation, tumor stages and nodal status, metastatic sites, the number of metastases and involved metastatic sites, treatment of the primary tumor, treatment of oligometastasis, response rate, OS, and progression-free survival (PFS) were evaluated.

The diagnosis of NSCLC was confirmed based on histopathology and staged according to the eighth edition of the TNM staging of the International Association for the Study of Lung Cancer [15]. Radiological staging includes computed tomography (CT) scan and magnetic resonance imaging (MRI). In addition, PET/CT was added as appropriate to define the treatment strategy, to assess systemic treatment response, and to help identify the patient cohort for which aggressive local treatment is more appropriate.

The metastatic regions of the disease (brain, bone, adrenal gland, liver) were determined and, after that a number of metastases ≤ 5 (3 for brain metastases) was included. Surgery and/or curative RT for the primary tumor and metastatic site were considered radical treatment. In addition, chemotherapy, immunotherapy and targeted therapies were recorded for the treatment of primary tumors. All treatment decisions were made by a multidisciplinary oncology council.

Patients who had not undergone local ablative treatments for oligometastasis, those with life-threatening brain metastases and those with visceral crisis were excluded from analysis. Furthermore, patients with a history of other malignancies, except for basal cell skin carcinoma or in situ carcinoma of the uterine cervix, were not included.

The Local Ethics Committee of İstanbul Medipol University approved the study (decision number: E-10840082-771-02-2492).

The treatment responses, including partial response (PR) and complete response (CR), stable disease (SD) and progressive disease (PD), as well as the final objective response rate (ORR: PR and CR) were evaluated according to the RECIST 1.1.

### 2.1. Statistical analysis

Statistical analysis was made using SPSS Statistics for Windows, version 22.0. Descriptive statistics were used to summarize baseline characteristics. Survival analysis was done via Kaplan-Meier analysis and comparisons were made via a logrank test. PFS was defined as the time from diagnosis until disease progression or the date of death or loss in follow up. OS was described the time from diagnosis to the date of the patient’s death or loss in follow up. In addition, OS-2 was also defined as the time of first progression to the date of the patient’s death or loss in follow up. Univariate analysis was carried out to evaluate the significance of clinicopathological features as prognostic factors. Multivariate analysis with the Cox proportional hazards model was then performed in order to locate the independent prognostic factors for both PFS and OS. The 95% confidence interval (CI) was used to quantify the relationship between survival time and each independent factor. Multivariate p-values were used to characterize the independence of these factors. All p-values were two-sided, and p-values less than 0.05 were considered statistically significant.

## 3. Results

In this study, a total of 134 patients with oligometastatic NSCLC were included. Of these, 78.4% (n = 105) were men and 21.6% (n = 29) were female. Their median age was 59 years (range: 29–84). Most of the patients had been smokers at some point in their lives. Only 21.6% (n = 20) were never smoked. Histopathologically, 89 patients (66.4%) were defined as having adenocarcinoma, 35 (26.1%) as having squamous cell carcinoma and 10 (7.5%) patients had other histologies. The majority of patients were in ECOG performance status 0–1 (n = 126, 94%) and had no driver mutation (n = 108, 80.6%). According to the driver mutation status, EGFR (epidermal growth factor receptor) mutation was found in 15 patients (EGFR exon 19 del in 8 and EGFR 21 mut in 7 patients), ALK (anaplastic lymphoma kinase) in 7 patients, ROS-1 mutation in 3 patients, and BRAF in 1 patient. Initially 12.7% (n = 17) patients were classified as early stage, 14.2% (n = 14) as locally advanced and 73.1% (n = 98) as metastatic stage. Based on the treatment of the primary tumor, 36 patients (26.9%) underwent curative surgery, 19 (14.2%) received chemotherapy, and 73 (54.5%) were treated with chemoradiotherapy, while 1 (%0.7) received immunotherapy and 2 (1.4%) received targeted therapy. The most common metastatic site was the brain (n = 55, 41%), followed by bone (n = 29, 21.6%) and the adrenal gland (n = 16, 11.9%). While 58.2% of the patients had single site metastases, it was found that 15.7% of them had metastases in two sites and 26.1% also had metastases in 3 to 5 regions. The preferred treatments for oligometastatic lesions were SBRT in 72.4% of patients, surgery in 10.5%, and both SBRT and surgery in 17.1% of patients. Table 1 shows the baseline patient and tumor characteristics.

**Figure 1:**
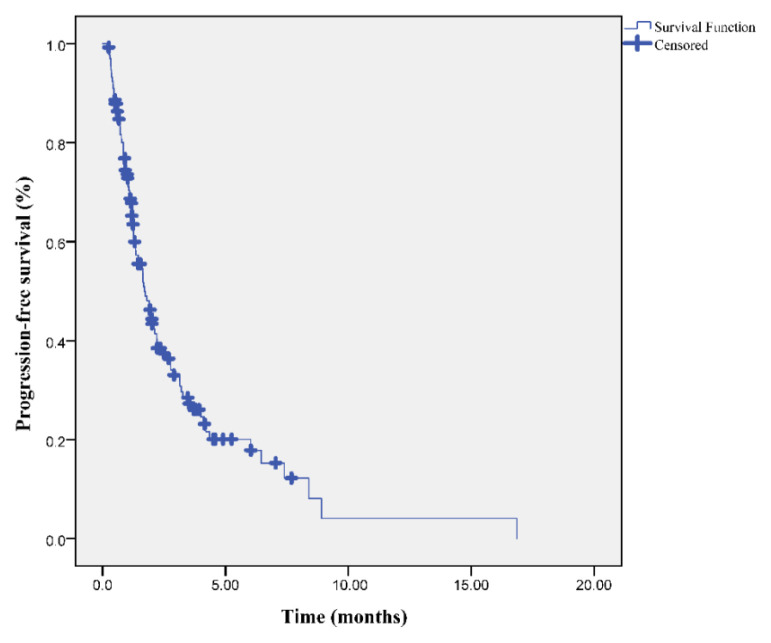
Progression-free survival curve in patients with oligometastatic NSCLC.

**Figure 2:**
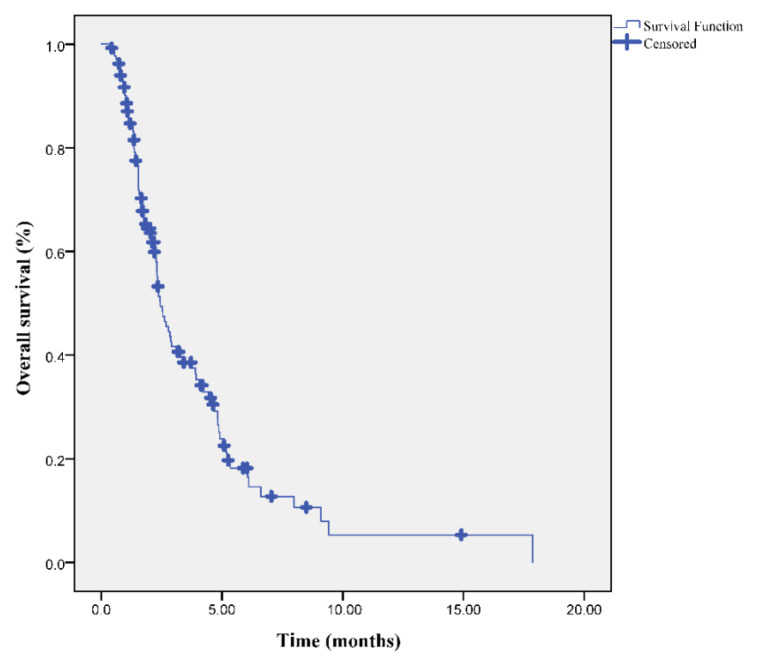
Overall survival curve in patients with oligometastatic NSCLC.

**Figure 3:**
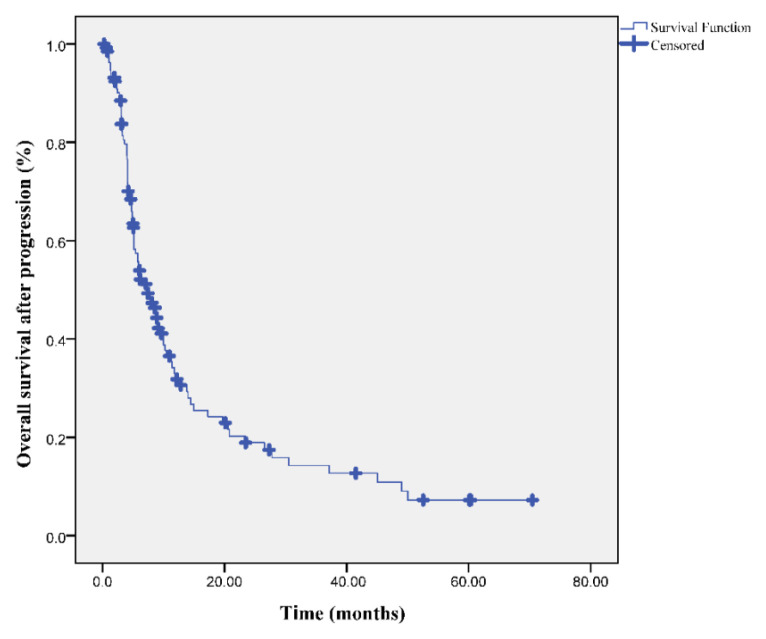
Overall survival-2 curve in patients with oligometastatic NSCLC after progression.

At the median follow up of 31.3 months (range: 9.5–48.5), the median PFS and OS times were 17 and 24.4 months, respectively. Moreover, OS-2 after progression was also 7.2 months (Figures 1–3).

In all patient cohort, univariate analysis for PFS revealed that >5% weight loss was a significant prognostic indicator (p = 0.041). The other significant prognostic factor was the presence of an oncodriver mutation. In other words, the median PFS time for patients with an oncodriver mutation was significantly better than those with a lack of driver mutation (31.5 vs. 14.4 months, p = 0.034). As expected, a significant correlation was found between stage at diagnosis and PFS (p = 0.027). Despite the T stage, significant correlation was detected between the N stage and PFS (p < 0.001). Furthermore, significant differences were also found with respect to histopathological type (p = 0.004), the type of treatment for the primary tumor (p = 0.001), metastatic sites (p < 0.001), and no. of metastases (p = 0.039). Median PFS time in patients with 3–4 metastases was worse than that in patients with one or two metastases (10.3 vs. 20.9 vs. 16.4 months, respectively). However, when the univariate analysis was performed for OS, we detected that the histopathologic type of tumor (p < 0.001), the presence of oncodriver mutation (p = 0.03), the N stage of the tumor (p = 0.005), the stage at diagnosis (p = 0.017), the metastatic sites (p < 0.001), the number of involved organs (p = 0.037) and the treatment of primary tumor (p = 0.001) were important prognostic factors. Multivariate analysis revealed a significant correlation between survival after progression and the histopathologic type of tumor (p > 0.001) and the presence of an oncodriver mutation (p = 0.005) (HR for survival after progression 1.36 and 0.41, respectively). In other words, the median OS interval for patients with adenocarcinoma histology was better than that for those with squamous cell carcinoma and NOS histologies (28.9 vs. 22 months). Similarly, median OS time was found to be worse compared to that in patients without oncodriver mutation (23.1 vs. 39.1 months, respectively). The results of univariate analyses for both PFS and OS are listed in Tables 2 and 3.

A multivariate analysis with the Cox proportional hazards model was performed in order to further evaluate all of the significant prognostic factors that were detected in the univariate analysis for patients with oligometastatic NSCLC. It showed that the presence of an oncodriver mutation (p = 0.006, HR: 0.42; 95% CI 0.23–0.78) and N stage (p = 0.003, HR: 1.71; 95% CI 1.20–2.42) were independent prognostic factors for PFS. On the other hand, the histopathologic type of tumor, the presence of oncodriver mutation and response to the initial treatment were found to be independent prognostic factors for OS by multivariate analysis (Tables 2 and 3).

Analyzes were performed again after excluding patients with driver-mutation due to the possibility of affecting the results. For PFS, the histopathologic type of tumor (p = 0.018), the N stage of the tumor (p < 0.001) the T stage of the tumor (p = 0.001), the stage at diagnosis (p = 0.004), the metastatic sites (p < 0.001), the number of involved organs (p= 0.037) and the treatment of primary tumor (p < 0.001), > 5% weight loss (p = 0.038) and no. of metastases (p = 0.027) were important prognostic factors. Univariate analysis for OS showed the histopathologic type of tumor (p < 0.001), the N stage of the tumor (p = 0.006), the T stage of the tumor (p = 0.01), the stage at diagnosis (p = 0.038), the metastatic sites (p < 0.001), the number of involved organs (p = 0.011) and the treatment of primary tumor (p < 0.001), > 5% weight loss (p = 0.007) and no. of metastases (p = 0.008) were found to be important prognostic features. Similarly, multivariate analysis was performed again after excluding patients with driver-mutation. It revealed that the N stage of the tumor (p = 0.002, HR:1.66, CI 95% 1.19–2.30) and the stage at diagnosis (p = 0.032, HR:1.43, CI 95% 1.03–2.00) for PFS and histopathologic type of tumor (p = 0.002, HR:1.38; CI 95% 1.12–1.69) and response to the initial treatment (p < 0.00, HR:1.61, CI 95% 1.26–2.06) for OS were independent prognostic factors.

After that, we analyzed prognostic factors in terms of OS-2 after first progression by the multivariate analysis. This analysis showed that histopathological type (p < 0.001, HR: 1.39; 95% CI 1.15–1.68) and the presence of oncodriver mutation (p = 0.02, HR:0.49; 95% CI 0.27–0.89) were found to be significantly independent prognostic factors, as was the number of metastasis (p = 0.035, HR:1.31; %95 CI 0.98–1.73). Table 4 shows the results of multivariate analysis for OS-2 after first progression. Similarly, when multivariate analysis was performed after excluding patients with driver mutation, it demonstrated that histopathological type (p < 0.001, HR: 1.52; 95% CI 1.25–1.85), number of metastases (p = 0.033, HR: 1.37; 95% CI 1.02–1.84) and > 5% weight loss (p = 0.044, HR: 1.62; CI 95% 0.94–2.78) were independent prognostic indicators for OS-2.

Treatment responses were assessed with RECIST 1.1. ORR was 86.1% with radical treatments for oligometastasis and primary tumors. The numbers of patients who achieved a response were as follows: 33 patients with CR, 69 patients with PR and 16 patients with SD (Table 5). Patients with good response (PR or CR) to the first-line treatments with radical treatments for oligometastasis and primary tumors oligometastasis had better OS compared to those with SD or PD (26.7 and 37.1 months vs. 17.2 and 13.4 months, respectively, p < 0.001).

## 4. Discussion

The main approach for oligometastatic cancer patients has not been well described yet. Surgery, SBRT, SRS, RFA or microwave ablation, which are known as locally ablative treatments, can be used for these patients [8]. The PFS benefit of local treatments was shown in two lung cancer, one prostate cancer, one colorectal cancer and one multiple histology studies [16–20]. To date, one prospectively randomized study has shown the improvement in PFS and OS in a small number of patients with oligometastatic NSCLC [21].

Having a nonadenocarcinoma histology, intracranial metastasis, or synchronous disease and being male were established as the risk factors for poorer prognosis in one retrospective study, which included 309 patients with oligometastatic cancer (≤5 metastasis) [22]. We evaluated the factors affecting OS and OS-2 separately in our study. Multivariate analysis showed that the histopathologic type of tumor, the presence of oncodriver mutation and response to the initial treatment were found to be significant predictive factors for OS. However, the histopathologic type, presence of oncodriver mutation and number of metastasis sites were predictive for OS-2. Our findings were thus compatible with the literature [22].

In retrospective studies, the contribution of local treatments in oligometastatic NSCLC has been demonstrated by evaluating lymph node status, tumor histology, thoracic disease bulk, ECOG PS, and number of metastatic sites [23, 24]. We found a significant relationship between weight loss, histopathologic type, presence of oncodriver mutation, metastatic sites, number of metastases, lymph node status and treatment of primary tumor and PFS in our study. While median PFS time could not be reached in those receiving immunotherapy and targeted therapy for primary tumor, it was <20 months in those receiving RT or chemotherapy. However, treatment strategy for oligometastasis was not a prognostic factor for PFS. This might be related to the relatively small sample size.

In one study, 23 patients with solitary metastasis NSCLC were treated with chemotherapy (mitomycin, vinblastine, and cisplatin) and resection with postoperative RT. The median OS time was 11 months, and 2 patients (9%) had a survival time of at least 5 years [25]. In one other prospective study that enrolled 39 oligometastatic NSCLC patients, the median PFS time was 12.1 months, and the median OS time was 13.1 months. There were no clinical features correlated with survival [26]. In our study, the median PFS, OS and OS-2 were 17, 24.4 and 7.2 months, respectively, at the median follow up time of 31.3 months. Today, newly targeted therapies, the development of immune check point inhibitors and improvement in RT techniques have prolonged survival in NSCLC patients [21, 22, 24]. This can be considered as one of the reasons for the difference in survival between our study and previous studies. The limitation of these trials and our study is the absence of a comparison arm [16–18].

In one prospective randomized study, those who responded well to front-line treatment were randomly assigned local treatment or observation/maintenance therapy. At the median follow up time of 12.39 months, median PFS time was 11.93 months in the locally treated arm and 3.9 months in the control arm, which corresponded to an HR for the locally treated group of 0.35 [21]. In our study, FDG PET-CT was used for the staging of almost all patients. However, in prospective study, 2% received PET/CT in the locally treated arm vs. 58% in the maintenance arm. The time to the appearance of a new lesion was longer among patients in the locally treated arm group (11.9 months vs. 5.7 months) [21]. Contrary to this study, the factors affecting OS and PFS were evaluated in our study with multivariate and univariate analysis due to the sufficient number of patients. In our study, 26.1% had 3–5 metastases and 15.7% had 2. However, another recent study enrolled mostly patients with less than 2 metastases [21]. In addition, in the prospective study, 27% of patients had brain metastases, while in our study it was 41%.

The main limitation of this study is the lack of a prospective, randomized-controlled study design with a relatively small sample size. Although our study was a retrospective study, the number of patients with worse prognostic characteristics was sufficient. Our study found better survival PFS and OS compared to the literature on oligometastatic disease [21]. Therefore, we have contributed to the literature by presenting the contribution of local treatment approaches to oligometastatic disease as a real-life experience.

Our study and the prospective studies in the literature included patients with different histology and molecular types. Although it is known that there is a difference in prognosis between molecular subtypes, a prospective study with stricter inclusion criteria was not possible due to cost. The multivariate analysis in our study showed that among the patients who progressed after first-line treatment, histopathological type (adenocarcinoma histology vs. squamous cell carcinoma or NOS histologies) was a significant prognostic factor in terms of OS-2 (p < 0.001 95% CI 1.15–1.68).

## 5. Conclusion

In conclusion, based on our real-life experience, we have shown that there is a significant relationship between good response to initial treatment and survival in oligometastatic disease, and also a significant relationship between survival after progression and histological type, presence of oncodriver mutation and number of metastases. We have also determined that in oligometastatic NSCLC patients, local ablative treatment modalities prolonged both OS and PFS and also delayed the OS-2. Prospective randomized trials are needed to further clarify the role of ablative therapy and survival benefit to all sites of disease in these patients.

## Figures and Tables

**Table 1:** Baseline patient and tumor characteristics.

Characteristics	N (%)
**Total patients**	134
**Age, years (median, range)**	59 (29–84)
≤60	76 (56.7)
>60	58 (43.3)
**Sex**	
Male	105 (78.4)
Female	29 (21.6)
**Smoking status**	
Never smoked	29 (21.6)
Exsmoker	71 (53)
Current smoker	34 (25.4)
**Weight loss (>%5)**	
Absent	79 (59)
Present	55 (41)
**Histopathological type**	
Adenocarcinoma	89 (66.4)
Squamous-cell carcinoma	35 (26.1)
Large-cell carcinoma	1 (0.7)
NOS	7 (5.2)
Others	2 (1.5)
**ECOG PS**	
0–1	126 (94)
2	8 (6)
**Oncodriver mutation**	
Absent	180 (80.6)
Present	26 (19.4)
**Tumor stage**	
T1	20 (14.9)
T2	62 (46.9)
T3	35 (26.1)
T4	17 (12.7)
**N stage**	
N0	30 (22.4)
N1	32 (23.9)
N2–3	72 (53.7)
**Stage at diagnosis**	
Early stage	17 (12.7)
Locally advanced	19 (14.2)
Metastatic	98 (73.1)
**Metastatic sites**	
Brain	55 (41)
Adrenal gland	16 (11.9)
Bone	29 (21.6)
Lung	13 (9.7)
Liver	2 (1.6)
Other	1 (0.8)
Multiple locations	18 (13.4)
**No. of metastases**	
1	78 (58.2)
2	21 (15.7)
3–5	35 (26.1)
**No. of involved organ**	
1	114 (85.1)
≥2	20 (14.9)
**Treatment of primary tumor**	
Surgery	36 (26.9)
Radiotherapy	3 (2.2)
Chemotherapy	19 (14.2)
Chemoradiotherapy	73 (54.5)
Immunotherapy	1 (0.7)
Targeted therapy	2 (1.4)
**Treatment of oligometastasis**	
SBRT	97 (72.4)
Surgery	14 (10.5)
SBRT and surgery	23 (17.1)

**SBRT:** stereotactic radiation therapy, **ECOG PS:** Eastern Cooperative Oncology Group Performance Status.

**Table 2:** Univariate and multivariate analysis for progression-free survival (PFS).

Features	Median PFS (month)	Univariate p-value	Multivariate p-value	HR 95% CI
**Age, years**		0.93		
≤60	16.7			
>60	17.3			
**Sex**		0.57		
Male	17.0			
Female	17.3			
**Smoking status**		0.125		
Never smoked	21.2			
Exsmoker	20.9			
Current smoker	12.1			
**Weight loss (>%5)**		**0.041**	0.99	1.01 (0.62–1.60)
Absent	21.2			
Present	12.5			
**Histopathological type**		**0.004**	0.085	1.19 (0.97–1.44)
Adenocarcinoma	20.3			
Squamous-cell carcinoma	10.5			
Large-cell carcinoma	NA			
NOS	9.1			
Others	NA			
**ECOG PS**		0.62		
0–1	17.9			
2	16.4			
**Oncodriver mutation**		**0.034**	**0.006**	**0.42 (0.23–0.78)**
Absent	14.4			
Present	31.5			
**Tumor stage**		0.77		
T1	22.1			
T2	15.5			
T3	17.9			
T4	13.0			
**N stage**		**<0.001**	**0.003**	**1.71 (1.20–2.42)**
N0	43.3			
N1	13.3			
N2–3	14.4			
**Stage at diagnosis**		**0.027**	0.17	1.25 (0.90–1.75)
Early stage	46.8			
Locally advanced	19.8			
Metastatic	13.0			
**Metastatic sites**		**<0.001**	0.55	0.96 (0.85–1.08)
Brain	19.8			
Adrenal gland	16.4			
Bone	14.4			
Lung	35.1			
Liver	13.0			
Other	6.2			
Multiple locations	3.4			
**No. of metastases**		**0.039**	0.67	0.93 (0.69–1.26)
1	20.9			
2	16.4			
3–5	10.3			
**No. of involved organ**		0.053	0.13	1.74 (0.83–3.63)
1	19.8			
≥2	13.0			
**Treatment of primary tumor**		**0.001**	0.56	0.92 (0.70–1.21)
Surgery	24.3			
Radiotherapy	18.4			
Chemotherapy	10.3			
Chemoradiotherapy	30.1			
Immunotherapy	NA			
Targeted therapy	NR			
**Treatment of oligometastasis**		0.78		
SBRT	17.3			
Surgery	16.4			
SBRT and surgery	18.8			

**SBRT:** stereotactic radiation therapy, **ECOG PS:** Eastern Cooperative Oncology Group Performance Status, **NA:** not applicable, **NR**: not reached, **HR**: hazard ratio, **CI**: confidence interval.

**Table 3:** Univariate and multivariate analysis for overall survival (OS).

Features	Median OS (month)	Univariate p-value	Multivariate p-value	HR 95% CI
**Age, years**				
≤60	25.4	0.83		
>60	23.6			
**Sex**		0.73		
Male	25.4			
Female	24.4			
**Smoking status**		0.07		
Never smoked	26.7			
Exsmoker	28.9			
Current smoker	20.5			
**Weight loss (>%5)**		**0.013**	0.53	1.16 (0.72–1.87)
Absent	31.4			
Present	22.6			
**Histopathological type**		**<0.001**	**0.002**	**1.36 (1.12–1.66)**
Adenocarcinoma	28.9			
Squamous-cell carcinoma	22.0			
Large-cell carcinoma	NA			
NOS	22.2			
Others	7.4			
**ECOG PS**		0.94		
0–1	24.4			
2	23.6			
**Oncodriver mutation**		**0.03**	**0.005**	**0.41 (0.22–0.76)**
Absent	23.1			
Present	39.1			
**Tumor stage**		0.57		
T1	28.9			
T2	23.6			
T3	27.8			
T4	23.0			
**N stage**		**0.005**	0.07	1.36 (0.96–1.90)
N0	64.4			
N1	22.6			
N2–3	23.3			
**Stage at diagnosis**		**0.017**	0.16	1.26 (0.90–1.75)
Early stage	74.9			
Locally advanced	27.8			
Metastatic	22.2			
**Metastatic sites**		**<0.001**	0.37	0.94 (0.83–1.06)
Brain	24.4			
Adrenal gland	28.9			
Bone	23.3			
Lung	57.6			
Liver	17.9			
Other	10.6			
Multiple locations	5.5			
**No. of metastases**		**0.016**	0.53	1.10 (0.81–1.49)
1	28.9			
2	24.3			
3–5	18.1			
**No. of involved organ**		**0.037**	0.06	2.03 (0.97–4.27)
1	25.3			
≥2	20.5			
**Treatment of primary tumor**		**0.001**	0.73	1.04 (0.80–1.37)
Surgery	45.4			
Radiotherapy	51.6			
Chemotherapy	16.3			
Chemoradiotherapy	39.8			
Immunotherapy	NA			
Targeted therapy	41.9			
**Treatment of oligometastasis**		0.81		
SBRT	24.4			
Surgery	28.9			
SBRT and surgery	23.0			
**Response to initial treatment**		**<0.001**	**<0.001**	**1.71 (1.36–2.14)**
CR	37.1			
PR	26.7			
SD	17.2			
PD	13.4			

**SBRT:** stereotactic radiation therapy, **ECOG PS:** Eastern Cooperative Oncology Group Performance Status, **NA**: not applicable, **NR:** not reached, **HR:** hazard ratio, **CI:** confidence interval.

**Table 4:** Multivariate analysis for overall survival-2 (OS-2) after first progression.

Factors	p	HR	95% CI
**Weight loss (>%5)**	0.17	1.38	0.86–2.22
**Histopathological type**	<0.001	1.39	1.15–1.68
**Oncodriver mutation**	0.02	0.49	0.27–0.89
**N stage**	0.81	1.03	0.78–1.36
**Metastatic sites**	0.87	0.99	0.89–1.10
**No. of metastases**	0.035	1.31	0.98–1.73
**No. of involved organ**	0.12	1.65	0.86–3.18
**Treatment of oligometastasis**	0.46	1.08	0.86–1.36

**HR**: hazard ratio, **CI**: confidence interval.

**Table 5:** Response rates according to the RECIST 1.1 in patients with oligometastatic NSCLC.

Response rate	n (%)
Complete response	33 (34.6)
Partial response	69 (51.5)
Stable disease	16 (11.9)
Progressive disease	16 (11.9)
Objective response rate (CR+PR)	102 (86.1)

**CR:** complete response, **PR:** partial response, **NSCLC:** nonsmall cell lung cancer.
